# Deformity angular ratio is associated with neuromonitoring changes without a vertebral column resection: spinal deformity is more influential than type of surgery

**DOI:** 10.1007/s43390-023-00669-y

**Published:** 2023-03-17

**Authors:** Kenneth D. Illingworth, Ali A. Siddiqui, David L. Skaggs, Lindsay M. Andras

**Affiliations:** 1 Department of Orthopaedics, Cedars-Sinai Medical Center, Los Angeles, CA USA; 2 Jackie and Gene Autry Orthopedic Center, Children’s Hospital Los Angeles, 4650 Sunset Blvd, MS#69, Los Angeles, CA 90027 USA; 3 Keck School of Medicine of University of Southern California, Los Angeles, CA USA; 4 University of FL College of Medicine—Jacksonville, Jacksonville, FL USA

**Keywords:** Scoliosis, Kyphosis, Posterior spinal fusion, Vertebral column resection, Complications, Neurologic deficit

## Abstract

**Purpose:**

The purpose of this study is to investigate the effect of the deformity angular ratio (DAR) on intra-operative neuromonitoring (IONM) signal changes during posterior spinal fusion (PSF) without vertebral column resection (VCR).

**Methods:**

Retrospective review of severe pediatric spinal deformity patients treated with PSF without VCR or three-column osteotomy from 2008 to 2018. Exclusion criteria were prior instrumentation, lack of IONM, and incomplete radiographic data. Coronal DAR (C-DAR), sagittal DAR (S-DAR), and total DAR (T-DAR) were calculated and compared between patients with IONM signal loss and those without.

**Results:**

Two hundred and fifty-three patients met inclusion criteria. Forty-seven of two hundred and fifty-three (19%) patients had IONM signal loss. Intra-operative wake-up test was performed in seven cases; three of seven (43%) had a neurological deficit on wake-up test. All neurological deficits resolved at a mean of 41 days postop. IONM loss was associated with increased kyphosis (*p* = 0.003) and was not associated with Cobb angle (*p* = 0.16). S-DAR (*p* = 0.03) and T-DAR (*p* = 0.005) were associated with IONM signal loss but C-DAR was not (*p* = 0.06). Increased incidence of IONM signal loss was seen with S-DAR > 7 (*p* = 0.02) or T-DAR > 27 (*p* = 0.02). Twenty-four of ninety-two (26%) patients with S-DAR > 7 had IONM signal loss compared to twenty-three of one hundred and sixty-one (14%) with S-DAR ≤ 7 (OR, 2.1; 95% CI, 1.1–4.0). Seven of sixteen (44%) patients with T-DAR > 27 had signal loss compared to forty of two hundred and thirty-seven (17%) patients with T-DAR ≤ 27 (OR, 3.8; 95% CI, 1.3–10.9).

**Conclusion:**

Patients with S-DAR > 7 or T-DAR > 27 have a higher risk of IONM loss during pediatric PSF even in the absence of a VCR or three-column osteotomies.

**Level of evidence** II.

## Introduction

Spinal cord injury is an infrequent yet feared complication of spinal deformity surgery [1–4]. Intra-operative neuromonitoring (IONM) is used to mitigate the risk of iatrogenic neurologic injury and allow for continuous assessment of the patients’ neurologic function, providing real-time warnings for potential spinal cord injury [3, 5]. Changes in IONM signals have been found to have high sensitivity and specificity for identifying patients with neurologic injury [1, 2, 5, 6].

The deformity angular ratio (DAR), defined as the curve magnitude per level of spinal deformity, is a combined measure of both deformity magnitude and sharpness popularized by Wang et al. in 2016; they reported on the DAR as a tool for evaluating the risk of neurologic injury in patients with severe spinal deformity undergoing vertebral column resection (VCR) [3]. Past studies found that increased DAR is positively correlated with elevated risk of IONM signal loss and post-operative neurologic deficit during VCR and three-column osteotomies in both the pediatric and adult populations [3, 7–9]. However, there is no information in the literature on the association between DAR and the incidence of IONM signal loss in pediatric spinal deformity patients treated with posterior spinal fusion (PSF) without a VCR or three-column osteotomy. The purpose of this study was to investigate the effect of DAR on IONM changes in patients with severe pediatric spinal deformity undergoing PSF without a VCR or three-column osteotomy.

## Materials and methods

Approval from the Institutional Review Board was obtained, and a retrospective case series review was conducted on consecutive spinal deformity patients that underwent PSF between October 2008 and February 2018 from a single tertiary children’s hospital. Inclusion criteria were any patient undergoing primary PSF for diagnosis of spinal deformity with major curve angle ≥ 70°, pre- and post-operative radiographs and documentation of intra-operative IONM. Patients with a curve ≥ 70° were chosen as these curves represented larger, more severe curves. All spinal deformity patients were included regardless of primary diagnosis or curve characteristics. Exclusion criteria were treatment with VCR or three-column osteotomy and prior spine surgery with or without spinal instrumentation. Any patient who had PSF for diagnosis other than primary spinal deformity, such as trauma/tumor, were excluded. Medical charts were reviewed for age, sex, scoliosis etiology, deformity, surgical technique, levels of instrumentations, IONM results, estimated blood loss (EBL), surgical time, and complications. Radiographs were reviewed for pre-operative and post-operative Cobb angle, kyphosis, and DAR. Coronal, sagittal, and total DAR were calculated as defined by Wang et al. [3]. Coronal DAR (C-DAR) was equal to the coronal Cobb angle divided by number of involved vertebral levels. Sagittal DAR (S-DAR) was calculated as the maximum kyphosis divided by number of involved vertebral levels. Total DAR (T-DAR) was defined as the sum of C-DAR and S-DAR. Loss of IONM signals was defined as decrease in amplitude of greater than 50% in somatosensory evoked potentials (SSEPs) and/or trans-cranial motor evoked potentials (MEPs) as documented by the neurophysiologist technician and/or attending neurophysiologist.

Comparisons were performed between patients with IONM signal loss and those without IONM signal loss. The groups were analyzed for differences in demographics, scoliosis etiology, pre-operative Cobb angle, kyphosis, C-DAR, S-DAR, and T-DAR. Statistical analyses were performed using STATA software version 14 (StataCorp LLC; College Station, TX). Chi-squared test, Fisher’s exact test, and student’s *t* test were used to evaluate for differences between groups. Statistical significance was defined as *p* < 0.05.

## Results

There were 253 patients that met inclusion criteria. Patient demographics are listed in Table 1. Mean age was 13.7 ± 3 and 62% of patients were female. Patient etiologies are listed in Table 2 and included neuromuscular (*n* = 100), idiopathic (*n* = 87), syndromic (*n* = 25), congenital (*n* = 22), and Scheuermann’s kyphosis (*n* = 19). Sixty-five percent (165/253) of patients were diagnosed with scoliosis, twenty-two percent (56/253) with kyphoscoliosis (> 70 degrees of deformity in both coronal and sagittal plane), and thirteen percent (32/253) with kyphosis. Mean pre-operative coronal Cobb angle was 82 ± 26 (0–147) degrees and mean pre-operative sagittal kyphosis was 55 ± 27 (3–141) degrees. The average number of vertebral levels fused was 13 ± 2 (3–18). Ponte osteotomies were performed in 195/253 patients; in those 195 patients, the mean number of Ponte osteotomies was 5 ± 2 (1–12).

**Table 1:** Patient demographics

	No loss of IONM signals (*n* = 206)	Loss of IONM signals (*n* = 47)
Age	13.9 ± 2.9	12.8 ± 3.1*
Sex male/female (%male)	71/135 (34.5%)	25/22 (53.2%)*

*p < 0.05

**Table 2:** Etiology

	No loss of IONM signals (*n* = 206)	Loss of IONM signals (*n* = 47)	% Loss of signal
Idiopathic	71	16	18.4%
Neuromuscular	82	18	18.0%
Congenital	18	4	18.2%
Syndromic	17	8	32.0%
Scheuermann’s kyphosis	18	1	5.3%*

*The rate of signal loss was significantly lower for Scheuermann’s kyphosis compared with idiopathic, neuromuscular, and syndromic (*p* < 0.05)

**Fig. 1:**
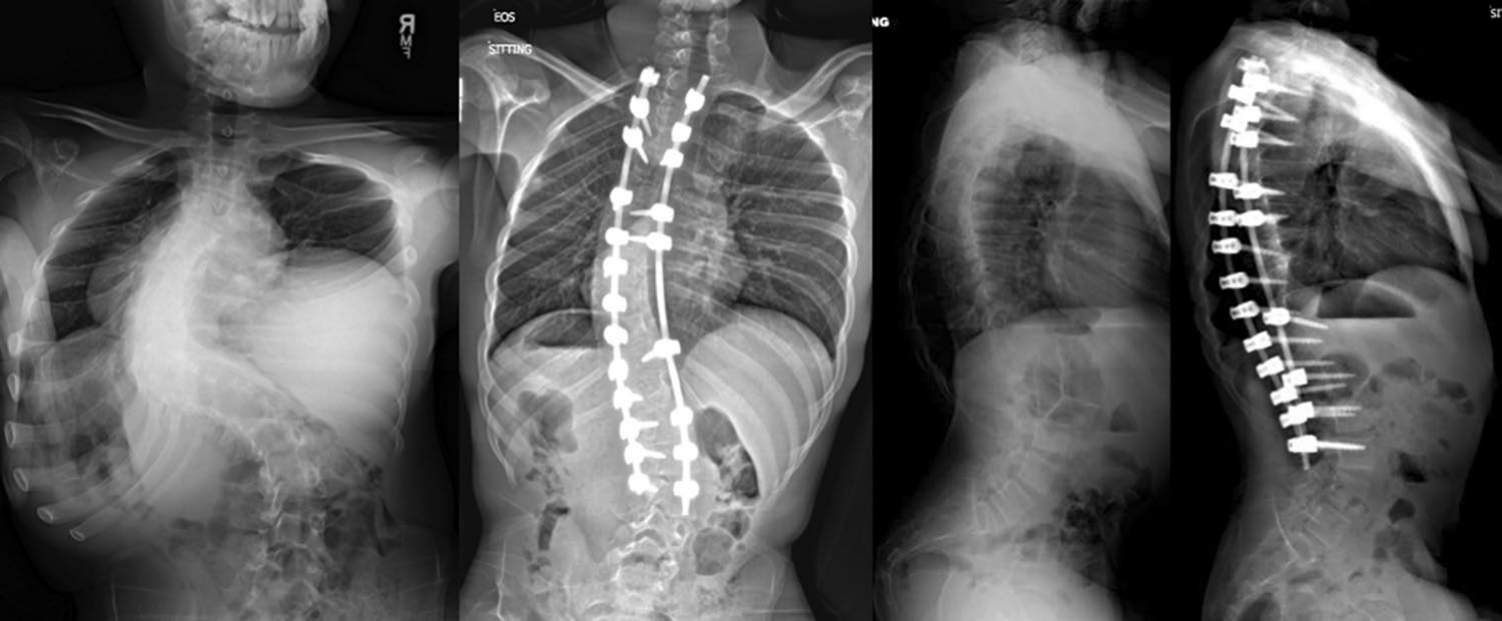
Thirteen-year-old male with neuromuscular scoliosis (who is ambulatory) measuring 106 degrees with C-DAR = 13, S-DAR = 9, and T-DAR = 22. This patient underwent PSF from T2-L3 without vertebral column resection or three-column osteotomy. This patient had loss of IONM signals but did not develop a post-operative neurological deficit. Radiographs at 6 months postop demonstrate significant deformity correction

**Fig. 2:**
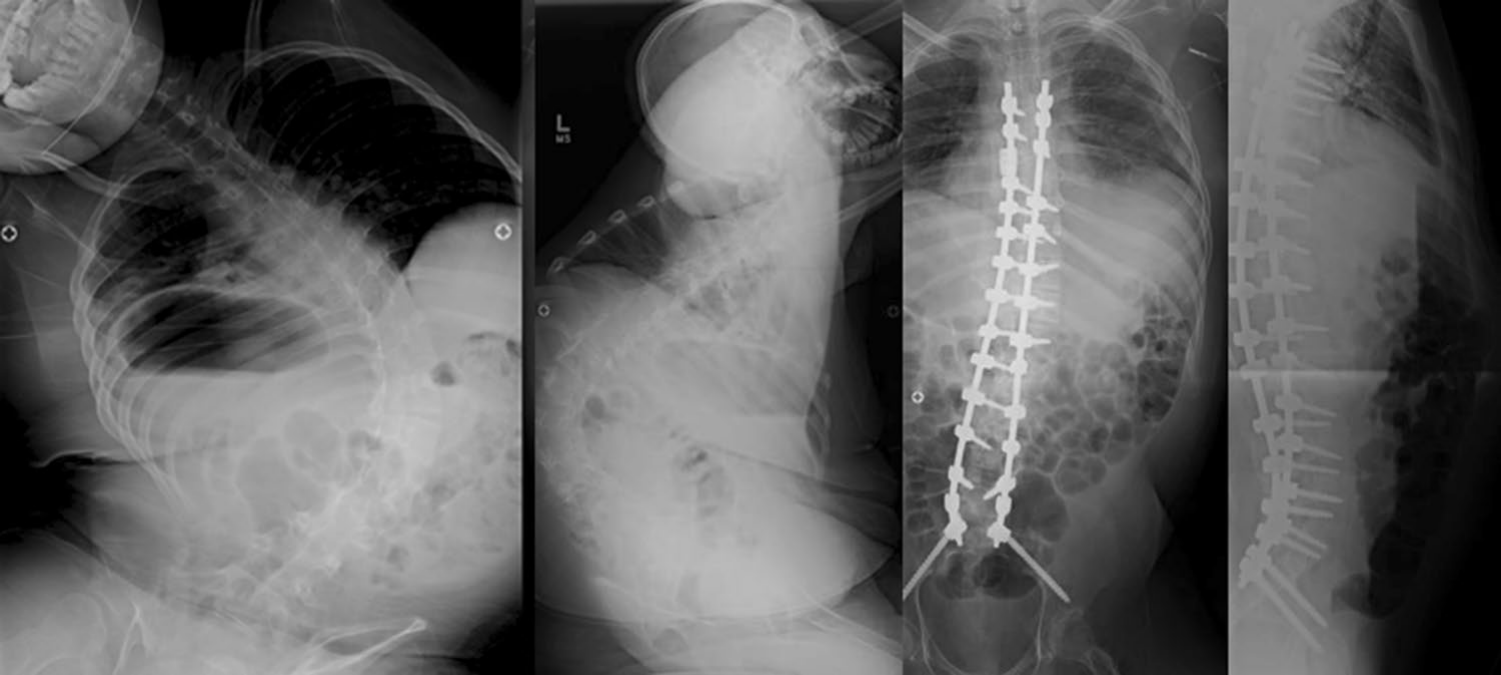
Fifteen-year-old male with a coronal curve magnitude 111-degree neuromuscular scoliosis secondary to Duchenne’s muscular dystrophy and C-DAR = 11, S-DAR, 10, T-DAR = 21. This patient was treated with PSF from T5-pelvis without vertebral column resection or three-column osteotomy. IONM signals remained stable throughout surgery and there were no instances of IONM signal loss. Follow-up radiographs at 1-month postop reveal coronal correction of the spinal deformity

Loss of IONM signals occurred in 19% (47/253) of patients; 81% (206/253) of patients did not have IONM signal loss. IONM changes involved MEPs alone in 31 cases, combined MEPs and SSEPs in 12 cases, and SSEPs alone in 4 cases. Loss of signal occurred during correction in 23 cases, pedicle screw insertion/instrumentation in 8 cases, and other (positioning/dissection/traction/etc.) during 16 cases. Among the 47 patients with IONM signal loss, 79% (37/47) had subsequent return of IONM signals to baseline and 21% (10/47) had signal loss without intra-operative return to baseline. Intra-operative wake-up test was performed in seven cases due to IONM signal loss. Three of these patients had a neurological deficit on wake-up test. All three neurological deficits occurred in patients with IONM signal loss without return to baseline who underwent intra-operative wake-up test. The incidence of neurological deficits among all patients was 1% (3/253). The observed neurological deficits in this series were paralysis in the bilateral lower extremities (two cases) and paralysis of the left lower extremity (one case). All deficits fully resolved at a mean of 41 days postop (range: 0–41 days). No patients developed a permanent neurological deficit. Of the 47 patients with IONM signal loss, 27 had a cervical/thoracic/lumbar magnetic resonance imaging study, of which only 3 had evidence of pre-existing neural axis abnormality (foramen/spinal stenosis with achondroplasia, thoracic syrinx, cervical syrinx) (Figs. 1, 2).

Patients with IONM signal loss were slightly younger at index surgery (12 vs 13 years, *p* = 0.03) and higher percentage of males (52%) (Table 1). Patients with Scheuermann’s kyphosis had lower incidence of IONM signal loss compared to idiopathic, neuromuscular, and syndromic (*p* < 0.05) (Table 2). IONM signal loss had longer surgical time (5.4 vs 6.2 h, *p* = 0.01) compared to those without IONM signal loss and there was no difference between groups in the mean number of vertebral levels fused (*p* = 0.12) or number of Ponte osteotomies (*p* = 0.07). Mean Cobb angle, kyphosis, C-DAR, S-DAR, and T-DAR of both groups are described in Table 3. IONM signal loss was associated with increased sagittal Cobb angle (*p* = 0.003) and was not associated with coronal Cobb angle (*p* = 0.16). Increased S-DAR (*p* = 0.03) and T-DAR (*p* = 0.005) were associated with IONM signal loss while C-DAR was not (*p* = 0.06) (Table 2). Increased risk of IONM loss was seen with S-DAR > 7 (*p* = 0.02) or T-DAR > 27 (*p* = 0.02). Among patients with S-DAR > 7, the incidence of IONM signal loss was 26% (24/92) compared to 14% (23/161) in patients with S-DAR ≤ 7 (OR, 2.1; 95% CI, 1.1–4.0). In patients with T-DAR > 27, 44% (7/16) had IONM signal loss compared to 17% (40/237) of patients with T-DAR ≤ 27 (OR, 3.8; 95% CI, 1.3–10.9) (Table 4).

**Table 3:** Comparison of deformity magnitude between PSF patients with vs without intra-operative neuromonitoring signal loss

Parameter	IONM signal loss (*n* = 47)	No IONM signal loss (*n* = 206)	*p* value
Sagittal kyphosis	64 ± 30	51 ± 30	0.003*
Coronal Cobb angle	86 ± 33	80 ± 24	0.16
Sagittal DAR	8 ± 4	6 ± 4	0.03*
Coronal DAR	12 ± 11	11 ± 4	0.06
Total DAR	20 ± 6	17 ± 5	0.005*

*p < 0.05

**Table 4:** Radiographic parameters predictive of intra-operative neuromonitoring signal loss

Parameter	Incidence of IONM signal loss	*p* value
S-DAR > 7	26% (24/92)	0.02*
S-DAR < 7	14% (23/161)	
T-DAR > 27	44% (7/16)	0.02*
T-DAR < 27	17% (40/237)	

*p < 0.05

## Discussion

This study was the first to examine the effect of DAR on the risk of IONM changes in pediatric spinal deformity patients treated with PSF without a VCR or three-column osteotomy. This study also represents the largest series analyzing the effect of DAR on the risk of IONM signal loss during any type of spinal deformity surgery. Prior authors reported that increased S-DAR and T-DAR are predictive of IONM signal loss among patients undergoing VCR or three-column osteotomy [3, 7, 8]. Our findings show that among pediatric spinal deformity patients undergoing PSF, increased S-DAR and T-DAR are associated with a higher rate of IONM signal loss even in the absence of a VCR or three-column osteotomies.

The odds ratio of IONM signal loss during PSF without VCR or three-column osteotomy in patients with T-DAR > 27 than in those with T-DAR ≤ 27 was 3.8 (*p* = 0.02). This demonstrates that T-DAR is predictive of IONM changes during spinal surgery, and this has been corroborated by previous authors [3, 7, 8]. However, all prior studies included patients treated with VCR or three-column osteotomies. Wang et al. identified the threshold of T-DAR ≥ 25 as being associated with a significantly higher risk of IONM signal loss during VCR; they found that patients with T-DAR ≥ 25 had a 41% incidence of signal loss [3]. Alternatively, our study found that patients undergoing PSF with T-DAR > 27 had a 44% incidence of IONM signal loss (*p* = 0.02). Lewis et al. modeled the positively correlated relationship between increasing T-DAR and estimated probability of IONM signal loss during surgery for severe spinal deformity [7]. According to their model, a patient with T-DAR > 27 has at least a 40% probability of having IONM signal loss during three-column osteotomy; this is similar to the 44% incidence of IONM signal loss during PSF seen in our study [7]. Additionally, the incidence of IONM signal loss in our patients overall—who solely underwent PSF without VCR—was 19% compared to the 20.5% incidence during VCR reported by Wang et al. [3]. The comparable incidences of neuromonitoring changes coupled with the association between high T-DAR and IONM signal loss seen in both our PSF patients and in VCR/three-column osteotomy patients in the literature suggest that T-DAR is predictive of IONM signal loss in pediatric spinal deformity surgery regardless of the surgical technique.

Our study also found that PSF patients with S-DAR > 7 had a significantly higher risk of IONM signal loss than those with S-DAR ≤ 7 (26% vs 14%; *p* = 0.02). Both Lenke et al. and Kim et al. similarly reported that severe pre-operative deformity in the sagittal plane is an important risk factor for neurologic complications during VCR [10, 11]. Past authors also concluded that elevated S-DAR is a significant risk factor for IONM signal loss during spinal surgery; Lee et al. additionally found that higher S-DAR was associated with a greater likelihood of any type of complication during VCR [3, 7, 8]. The correlation between elevated S-DAR and IONM signal loss during VCR, three-column osteotomies, and PSF alone further supports that the physical characteristics of the deformity may have greater influence on the risk of neuromonitoring changes than the type of surgery.

Interestingly, the role of the C-DAR in predicting IONM changes still appears debatable. There are conflicting reports in the literature regarding the utility of the C-DAR for assessing the risk of IONM signal loss during spinal surgery. In the original Wang et al. study of DAR, patients with C-DAR > 10 had a significantly higher incidence of IONM signal loss during VCR compared to those with C-DAR < 10 (32.9% vs 12.3%) [3]. Additional studies on VCR and three-column osteotomy patients by Lee et al. and Lewis et al., respectively, instead concluded that increased C-DAR does not have an effect on the incidence of signal loss [7, 8]. Our study included 253 PSF patients and found the C-DAR was not correlated with the incidence of IONM signal loss (*p* = 0.06), although it approached statistical significance. Although our series is the largest to date reporting on the role of DAR in IONM changes, it is possible that it was underpowered from the perspective of C-DAR. Additional investigation of C-DAR and its association (or lack thereof) with IONM changes is warranted.

This study is limited by its retrospective nature. In particular, the variable IONM techniques and definitions of IONM signal loss among different institutions is a challenge when comparing our results to those in the literature [5]. Additionally, spinal deformity centers with a high volume of these procedures, such as the center in this study, may have established protocols and dedicated, experienced spine anesthesiologists resulting in precautions that lower the overall incidence of signal changes even in these severe deformities. Furthermore, spinal deformities result from heterogeneous etiologies which may have a confounding effect both on IONM and on surgical outcomes. Syndromic patients in our study had a nearly twofold increase in IONM signal loss compared to idiopathic/neuromuscular/congenital (32% vs 18%); however, the syndromic group was likely underpowered to detect a significant difference and further analysis is warranted. In addition, spinal deformities can be associated with variable rates of neural axis abnormalities, which can possibly impact the rate of IONM changes. We did not specifically evaluate magnetic resonance imaging for all patients to be able to interpret these variables impact on our results. The culture at our institution has been a high threshold for VCR and three-column osteotomies for even severe/rigid curves. Therefore, there was no retrospective analysis on curve flexibility which other institutions might use to determine the type of osteotomies performed.

While our study found that IONM signal loss may occur at a similar rate during either PSF or VCR/three-column osteotomy, the incidence of permanent neurological deficit was 0% in our study compared to 6–13% after VCR/three-column as reported in the literature [12, 13]. Thus, our findings suggest that while there is a similar incidence of IONM changes between surgical techniques, it is important to note that the incidence of actual permanent neurological deficits is greater after VCR/three-column osteotomies. This difference may be related to the higher spine instability and more limited management options for IONM changes during VCR/three-column osteotomy.

This study supports that the DAR is a useful tool for predicting the risk of IONM signal loss during PSF in pediatric spinal deformity patients. Although past studies report that VCR more frequently results in complications than alternative surgical techniques, our study found that elevated S-DAR and T-DAR are associated with an increased likelihood of IONM changes during PSF without a VCR or three-column osteotomy. Spine surgeons should recognize this novel finding, as it suggests that the severity of the deformity, rather than specific surgery, may be the more influential contributor to the risk of IONM changes and potential for intra-operative neurologic injury.

## Data Availability

All figures in this manuscript are reproduced with permission of Jackie and Gene Autry Orthopedic Center, Children’s Hospital Los Angeles.
